# Estimation of spike initiation zone and synaptic input parameters of a *Drosophila* motoneuron using a morphologically reconstructed model

**DOI:** 10.1186/1471-2202-15-S1-P65

**Published:** 2014-07-21

**Authors:** Cengiz Günay, Astrid A Prinz

**Affiliations:** 1 Dept. Biology, Emory University, Atlanta, Georgia 30322, USA; 2 Dept. Math and Computer Sci., Emory University, Atlanta, Georgia 30322, USA

## 

The fruitfly *Drosophila* melanogaster has recently emerged as a powerful model for studying neuronal circuit development and function through identified neurons. A major obstacle to these studies has been a lack of computational models of these neurons for generating and testing hypotheses with fast-throughput simulations. Here, we constructed a computational model of an identified *Drosophila* motoneuron, aCC/MN1-Ib, from its recorded biophysical and anatomical properties [1]. Constructing this model required estimating the location and properties of distal spike-generating Na+ and K+ channels. The effects of channel location were simulated by including anatomical details in the model through a morphological reconstruction. By simulating a match to the observed in vivo electrophysiological characteristics, we predict the anatomical location of the spike initiation zone (SIZ) to be distal on aCC’s primary axon [2]. The complex morphology of the neuron effectively filters fast activating ionic currents which occur at the SIZ but are recoded from the soma *in vivo*. In particular, the transient component of the voltage-activated sodium current is extensively filtered relative to the slower persistent component. Input-output behavior in a broader network context was also predicted [2]: about 20 miniature excitatory potentials are required for initiating an action potential and about 200 individual potentials underlie evoked spontaneous rhythmic currents (SRC) in this neuron. Our model of aCC, which is the first morphologically-realistic active model of an identified *Drosophila* motoneuron, provides a template for further investigation of neuronal function in health and disease.
